# Digital Literacy and Food Consumption Structure: Evidence from Rural China

**DOI:** 10.3390/nu17132207

**Published:** 2025-07-02

**Authors:** Yanling Xiong, Yuchan Lin, Sihui Zhang, Tianyang Xing, Xiaowei Wen

**Affiliations:** 1 College of Economics and Management, Suzhou Polytechnic Institute of Agriculture, Suzhou 215008, China; 2 College of Economics and Management, South China Agricultural University, Guangzhou 510642, China; 3 Guangdong Research Center for Rural Policy, Guangzhou 510642, China; 4 College of Economy and Trade, Zhongkai University of Agriculture and Engineering, Guangzhou 510225, China

**Keywords:** digital literacy, rural residents, food consumption structure, awareness of dietary health, awareness of food safety

## Abstract

**Background/Objectives:** Healthy diets and proper nutrition are fundamental for human survival. With economic development and rising incomes, the food consumption structure of rural residents in China has noticeably changed. However, substantial disparities still exist in the quality of food consumption between urban and rural areas, and the dietary structure of rural populations remains imbalanced. **Methods:** This study uses 2020 China Rural Revitalization Survey (CRRS) samples of rural residents for analysis since it asked residents questions about their digital literacy and food consumption. A total of 2827 valid rural resident samples were finally obtained, and the participants had a mean age of 54.844 years. This study employs the ordinary least squares (OLS) model and the two-stage least squares (2SLS) method to examine the impact of digital literacy on the food consumption structure of rural households and its underlying mechanisms. **Results:** Based on the regression analyses, digital literacy significantly improves the food consumption structure of rural residents (*p* < 0.05). Heterogeneity analysis shows that at the regional level, digital literacy has a stronger impact on the food consumption structure of rural residents in southern China (β = −153.255, *p* < 0.05); at the individual level, its impact is more pronounced among rural residents with lower educational attainment (β = −427.506, *p* < 0.01) and among middle-aged and elderly populations (β = −212.705, *p* < 0.05). The mechanism analysis reveals that digital literacy can enhance the food consumption structure of rural residents by increasing their awareness of dietary health and food safety. **Conclusions:** These findings highlight the necessity of integrating the optimization of food consumption structures with enhancements in digital literacy into policy-making and provides valuable insights for developing policies aimed at improving the nutritional health of rural residents.

## 1. Introduction

The diet quality divergence (DQD) index refers to the gap between actual dietary patterns and recommended standards. A significant DQD index may increase the risk of chronic diseases, lead to malnutrition or overnutrition, and consequently bring about higher healthcare costs. Over the past forty years, China has implemented a range of measures to improve the food consumption structure and nutritional status of Chinese residents [1]. Since the proposal and implementation of an all-encompassing approach to food, China’s food consumption structure has been significantly optimized. This is due to an increase in food supply capacity and improvements in residents’ income and spending power. As a result, nutritional health levels have steadily improved, and the public is becoming increasingly aware of the importance of balanced dietary nutrition. It has been shown that household food expenditure in China has significantly increased, food consumption volume has noticeably risen, dietary patterns have gradually diversified, and dietary quality has steadily improved [2]. However, the improvement in food consumption is not only reflected in higher spending but also in the optimization of the microstructure and levels of various types of consumption among residents [3]. Data from the National Bureau of Statistics and the Chinese Dietary Guidelines indicate that rural residents exhibit a more severe deviation in food consumption compared to urban residents [4,5]. Specifically, rural residents consume grains and livestock meat in excess of recommended maximums, while their intake of starchy foods, vegetables, and dairy products is insufficient. Moreover, there is a significant gap between the actual and recommended intake of vitamin A, vitamin C, and calcium among rural residents [1]. Thus, rural residents continue to confront various nutrition and health challenges due to imbalanced dietary structures [6].

With the rapid growth of the digital economy, the internet has developed at an accelerated pace, profoundly influencing rural production and daily life. It has become deeply integrated into various facets of society, the economy, and everyday life. Digital literacy refers to the ability of rural residents to effectively use digital devices or software to identify, access, integrate, utilize, evaluate, and create digital information and resources in a digital environment, thereby supporting their survival and development [7]. As an essential skill in modern society, digital literacy plays a critical role in enabling individuals to make informed and healthy eating choices and behaviors [8].

In light of this, China has made significant efforts to strengthen internet infrastructure and digital literacy in rural areas [9]. As internet penetration and digital literacy among residents has continued to rise, the food consumption structure and behaviors of rural residents have undergone notable changes, particularly reflected in three aspects: shifting consumption preferences, enhancing consumption experiences, and stabilizing consumption expectations [10,11,12,13,14]. In terms of food consumption structure, there has been a transition from merely “eating enough” to “eating well”. The food consumption of rural residents has become more diversified, moving beyond the traditional self-sufficient grain consumption model. Empirical evidence indicates that as internet penetration continues to rise, residents’ food consumption expenditures are also increasing, with the annual growth rate of per capita food, tobacco, and alcohol consumption in rural areas reaching 9.71% [5]. Furthermore, the improvements in digital literacy in rural areas have effectively unlocked the food consumption potential of rural residents, enabling them to make purchases online and enjoy shopping experiences similar to those of urban residents [11,15], resulting in a notable increase in food consumption levels.

It is evident that in the digital economy era, digital literacy has become a new driving force for enhancing the health and nutrition of the food consumption structure among rural residents. Therefore, it is recommended to strengthen the development of digital literacy among rural populations, enabling them to leverage digital technologies to optimize their food consumption patterns. However, the existing literature lacks empirical evidence directly demonstrating the impact of digital literacy on the food consumption structure of rural residents and its underlying transmission mechanisms. This study aims to address the following questions: Is the observed correlation between the development of digital literacy and the optimization of food consumption structures in rural areas merely a statistical coincidence, or does digital literacy play a crucial role in optimizing these consumption patterns?

To clarify this issue, this study utilizes microdata from the China Rural Revitalization Survey (CRRS) to examine the impact of digital literacy on the food consumption structure of rural residents. Moreover, our findings aim to offer valuable policy implications for improving the dietary structure and health outcomes of rural residents, thereby facilitating their better integration into the digital health era.

This study offers several significant contributions to the literature. First, while the majority of existing research has concentrated on the economic benefits of enhancing digital literacy, there is a lack of studies investigating its impact on the optimization of food consumption structures. This work seeks to expand and enrich research in this area. Second, regarding variable selection, existing studies often use food consumption expenditure as a variable, emphasizing the quantity of food consumption while neglecting its quality. This study introduces dietary quality indices (DQD) and other dietary balance metrics to quantify food consumption structures from both quantity and quality perspectives. Third, from a research perspective, previous studies on the mechanism of digital literacy and food consumption are often discussed in terms of income. This paper will reveal the micro-mechanism of digital literacy in promoting the optimization of the food consumption structure at the psychological level.

## 2. Literature Review and Theoretical Framework

### 2.1. Digital Literacy

The development of the digital economy has injected new vitality into the consumption patterns of rural residents, profoundly transforming their lifestyles. As the digital rural strategy is further advanced, the utilization of digital technologies among rural residents has significantly increased [16]. According to human capital theory, an individual’s knowledge, skills, and experience contribute to the formation of human capital, which helps them achieve their goals. In the era of the digital economy, digital literacy is increasingly recognized as a crucial component of human capital [17]. It refers to the comprehensive set of digital skills and knowledge that individuals develop or possess in their production and daily activities within a digital environment [18,19]. The existing literature on digital literacy has primarily discussed its effects on residents’ social production and lifestyle from multiple dimensions. Research has found that improving digital literacy helps individuals make informed decisions and is closely related to healthy lifestyles, thereby enhancing their quality of life [19,20,21,22]. These studies primarily focus on examining the impact of digital literacy on various health aspects among specific groups, including exercise, diet, physical health, mental health, psychological well-being, life satisfaction, and overall quality of life [22,23,24]. It is clear that establishing a balanced food consumption structure is an integral component of health management. As a core indicator of digital utilization among rural residents, digital literacy significantly impacts their consumption of food. Specifically, greater digital literacy can equip rural residents with the ability to access dietary health guidance, improve their capacity to search for and evaluate health information, expand channels for acquiring health knowledge, and optimize information dissemination [25].

Previous studies have evaluated digital literacy from various dimensions using comprehensive assessment indicators, in alignment with the concept of digital literacy and the research objectives. Some studies have developed self-assessment scales for digital literacy, enabling respondents to evaluate their own competencies [18]. Additionally, other studies employ interviews to assess whether respondents possess the requisite knowledge and skills for digital literacy [26]. The measurement dimensions will encompass digital technology capabilities (e.g., information processing, communication, content creation), accessibility to digital technology (e.g., the number of personal computers, mobile devices), mobile device proficiency (e.g., basic knowledge of mobile devices, communication, data storage, entertainment), and internet usage conditions (e.g., computer usage and knowledge, internet usage and knowledge) [27,28,29]. However, there is limited research directly linking digital literacy with food consumption structure and nutritional intake [30], let alone addressing the development of comprehensive indicators of digital literacy to explore its impact on diet quality [1]. Therefore, this article constructs an empirical analysis framework to elucidate the impact mechanisms of digital literacy on the food consumption structure of rural residents.

### 2.2. Theoretical Framework

The rapid development of the digital economy has had multiple positive effects on the consumer market. At the micro level, it has significantly enhanced both the convenience of shopping and the diversity of choices available to consumers. Furthermore, it has facilitated improvements in the competitive pricing mechanisms within the market, thereby increasing consumption efficiency and overall consumer satisfaction [31]. At the macro level, the digital economy has effectively stimulated an upgrade in household consumption by reducing costs and expanding the scale of consumption. More critically, it promotes the optimization and upgrading of industrial structures by catering to personalized demands and improving the consumer experience. This, in turn, exerts a substantial enabling effect on the upgrading of residents’ consumption patterns [32]. Digital literacy, as a medium for reaping the benefits of the digital economy, holds tremendous potential for improving population health and individual well-being. Specifically, as discussed in this article, regions with higher levels of digital literacy can expand channels for information access, reduce transaction costs associated with food consumption for rural residents, and increase the diversity of food consumption [15,33]. This ultimately helps address issues such as imbalanced diets and nutritional deficiencies among rural residents.

#### 2.2.1. The Impact of Digital Literacy on Food Consumption Structure

Building on the existing literature, this study defines the digital literacy of rural residents as a comprehensive system encompassing digital knowledge, digital awareness, and digital skills, which are developed and applied in both production and daily life practices within a digital context [18,19]. Specifically, this concept includes four dimensions: digital information skills, digital life skills, digital media application skills, and digital production skills. In general, the greater the digital information skills of rural residents, the more comprehensive their knowledge base for utilizing digital tools to collect, organize, and process information, and the stronger their basic operational skills for e-commerce, online healthcare, and utility payments. This, in turn, helps to overcome barriers to adopting digital technology and reduces the participation costs of healthy diets in the digital world. Rural residents with greater digital life skills are better equipped to select and utilize digital products related to food, healthcare, and daily services, benefiting from the consumption demonstration effect of online social networks [34]. Those with greater digital media application skills not only actively adopt new products and functions, such as e-commerce platforms, online diagnosis apps, and daily service apps, but also leverage their digital editing and information dissemination skills to engage in community communication and experience sharing, thereby continually enhancing their perceived benefits and actual utility of participating in the digital world. Finally, rural residents with greater digital production skills can, on one hand, optimize the efficiency of market information acquisition through e-commerce platforms, and on the other hand, use digital financial tools to alleviate capital constraints, significantly improving their food consumption ability [35].

As a fundamental skill for individuals in the digital era, digital literacy plays a crucial role in facilitating the adoption of digital technologies such as e-commerce and mobile payments in rural areas, reducing transaction costs for rural residents, and enhancing the convenience of food consumption. In comparison to traditional information channels, the internet serves as an important carrier of knowledge and information, facilitating the flow and integration of information and data resources, which broadens the sources of information available to rural residents, expands their cognitive horizons, and reduces search costs [36]. Furthermore, modern network and information technologies have enabled the development of an omnichannel distribution model. In contrast to the high-cost scale of offline supply, the internet allows consumers to directly experience the cost-effectiveness of goods and benefit from online shopping discounts, thereby directly reducing residents’ living costs [36]. Additionally, the convenience, security, awareness, and benefits of mobile payments provide people with greater financial freedom, lowering the transaction barriers for rural residents in online shopping and reducing transaction costs [30]. For example, Xue et al. found that the utilization of digital technology can lower transaction costs incurred by rural residents during sales and purchases, enhance economic returns, and subsequently increase expenditures on food consumption, leading to improved dietary quality within households [15]. This indicates that digital technology has effectively reduced the living costs for rural residents, thereby prompting further enhancement in the food consumption levels of their households.

Moreover, digital literacy can effectively bridge the gap between rural residents and market access, thereby providing greater food choice opportunities and enhancing dietary diversity. In comparison to traditional offline markets, online shopping overcomes logistical and informational bottlenecks, offering a wide array of product categories that meet the diverse needs of rural households [11]. Additionally, the internet facilitates direct and rapid connections between buyers and sellers, offering consumers in remote locations an equitable digital, contextual, and social shopping experience [14], thus enhancing the diversity and quality of food consumption among rural residents. Existing research has shown that the use of internet-connected devices, such as smartphones or computers, significantly improves food diversity and safety for rural households [37]. The study conducted by Xue et al. explicitly demonstrates that the use of the internet and other digital technologies has led to increased consumption of nutritious foods—such as eggs, seafood, and dairy products—among rural residents, consequently enhancing the quality of their diets [15]. It is evident that, against the backdrop of an increasingly rich array of digital life scenarios, including e-commerce, mobile payments, and the sharing economy, the food consumption patterns of rural residents will continue to be optimized as their digital literacy advances. Drawing from this theoretical analysis, this paper proposes the following research hypothesis:

**H1.**  *The improvement in digital literacy can promote the optimization of food consumption structures among rural residents.*

#### 2.2.2. Mechanisms of Digital Literacy in Relation to Food Consumption Structure

Digital literacy can significantly aid rural residents in seeking health-related information, thereby bridging the digital health gap and promoting the establishment of healthy eating habits. In practice, residents are increasingly leveraging the internet and other digital technologies to access health information [38]. Research across various contexts indicates that digital literacy plays a crucial role in raising health awareness and facilitating the adoption of healthy behaviors among individuals [22]. Those with higher levels of digital literacy are more likely to embrace a healthy lifestyle and tend to opt for nutritionally balanced diets [39,40]. König et al. investigated the relationships among digital literacy, information evaluation, information searching, and various health behaviors, finding that enhanced digital literacy boosts awareness of healthy eating, leading to higher consumption of fruits and vegetables [22].

The relationship between awareness and behavior has long been a central concern in various disciplines. It is widely acknowledged in academia that human behavioral choices result from both rational and irrational factors [41]. This indicates that both the subjective factors of the actor and the objective constraints they face are critical when explaining social behavior. The Health Belief Model (HBM) emphasizes that the most direct psychological factors influencing the public’s adoption of certain behaviors are beliefs or motivations [42,43,44]. Similarly, the optimization of food consumption patterns among rural residents is inevitably shaped by psychological awareness, specifically health and safety awareness. Therefore, this study categorizes residents’ food health awareness into two dimensions: dietary health awareness and food safety awareness [45,46]. According to the HBM, individuals who hold beliefs about disease and health are more likely to adopt healthy behaviors and modify risky behaviors, suggesting that food consumption awareness can lead to changes in the current food consumption structure.

Dietary health awareness serves as a key driving force for healthy eating, motivating individuals to adopt related behaviors aimed at improving or enhancing their physical health [47] and increasing demand for health-conscious, organic, and green foods [43]. Research indicates that individuals with higher levels of dietary health awareness are more attuned to the hazards or threats associated with food, more inclined to trust information that promotes dietary health, and more actively engaged in activities that support healthy eating [48,49]. Specifically, such individuals are more likely to choose fresh, unprocessed, or minimally processed foods, such as vegetables, fruits, whole grains, and healthy fats, while reducing their intake of high-sugar, high-fat, high-salt, and processed foods to mitigate health risks. Consequently, individuals with greater dietary health awareness are more likely to adopt dietary behaviors that prevent illness, adjust their dietary structures, and optimize their overall food consumption structures.

Food safety awareness pertains to a proactive attitude and behavioral consciousness regarding food safety, involving individuals’ recognition and prevention of potential risks in food sourcing, processing, storage, and consumption. Those with strong food safety awareness tend to adopt cautious attitudes and controlled behaviors in their food consumption decisions, prioritizing safety and health over taste and texture [50]. As the demand for safe food increases, so does the level of food consumption. Furthermore, more consumers are becoming aware that industrial food supply chains in an unrestricted market may lead to food safety and nutritional concerns [51]. In this context, perceptions of food quality play a significant role in shaping consumers’ purchasing decisions, dietary patterns, and food preparation practices. Concerned about food safety and its effects on personal health, residents are increasingly focused on whether food is organic, natural, free from additives, non-GMO, and devoid of chemical residues [52,53]. Therefore, food safety awareness also reflects the extent to which residents value dietary quality and health, influencing their food consumption structure. This paper proposes the following hypothesis:

**H2.**  *Digital literacy promotes the optimization of food consumption structures by enhancing rural residents’ awareness of dietary health and food safety.*

## 3. Methods

### 3.1. Data Sources

The dataset utilized in this paper is derived from the 2020 China Rural Revitalization Survey (CRRS) database, a large-scale national rural tracking survey initiated by the Rural Development Institute, Chinese Academy of Social Sciences. The data are accessible for research purposes only. Access is granted upon submission of a request through an institutional affiliation. The datasets are available upon reasonable request, with permission from the Rural Development Institute, Chinese Academy of Social Sciences.

#### 3.1.1. Participants

This study focuses on residents in rural areas of China. Taking into account the significant regional disparities in economic and social development across the country, the CRRS research team employed random sampling methods to select 10 provinces/autonomous regions, including Guangdong, Zhejiang, Shandong, Anhui, Henan, Heilongjiang, Guizhou, Sichuan, Shaanxi, and Ningxia. The sample covered 50 counties/cities/districts, 150 townships, and 300 villages, with household survey data collected through standardized one-on-one interviews [54]. Participation was entirely voluntary, with all participants fully informed about the purpose of the study, how their data would be used, and any potential risks, and all provided informed consent.

To ensure the integrity of the research, only valid questionnaires containing complete data were included in the final analysis. In accordance with the objectives of this study, variables such as basic demographic information, digital literacy, food consumption, household income, household health status, and the number of individuals eating in the household were selected. Irrelevant variables and missing observations were then excluded, resulting in a final sample of 2827 rural residents.

#### 3.1.2. Procedure

The research team employed a systematic random sampling approach, taking into account factors such as economic development, spatial distribution, and agricultural production, as outlined below. First, sample provinces were randomly selected based on the regional classification standards of the National Bureau of Statistics for the east, central, west, and northeast regions. This selection process considers each province’s economic development level, geographic location, and agricultural situation. Second, a systematic random sampling method was applied at the county level, using per capita GDP as a basis for selection, ensuring that the chosen counties/cities/districts are spatially representative at the provincial level. Third, the same sampling technique was used to select sample towns and villages, with selection based on the economic development levels of local towns and villages. Fourth, households were randomly selected using a simple random sampling method, with rosters provided by the village committees [54].

#### 3.1.3. Survey Implementation and Quality Control

The research team established 10 fieldwork teams to carry out surveys across different provinces. Each interview lasted approximately 1.5 h. The survey team mainly consists of graduate students (master’s or doctoral level) and senior undergraduates from various universities. To ensure the reliability of the collected data, it was a requirement that survey team members have prior experience in rural research, come from rural household backgrounds, or possess relevant academic qualifications in fields such as agricultural economics, sociology, or related disciplines. Before formal data collection, the research team conducted indoor training to familiarize the surveyors with the content and logical structure of the questionnaire, followed by outdoor training, which involved a preliminary survey in a selected village. During this phase, the team leader addressed any challenges or questions raised by team members. To maintain data quality, the research team imposed a limit on the number of questionnaires each member could collect per day (≤4). Furthermore, a robust quality control system was implemented, which includes three rounds of verification for each completed questionnaire: on-site verification, evening group reviews, and a final assessment conducted by the team leader. It is evident that the CRRS has implemented various measures to control both sampling and non-sampling errors, thereby enhancing the overall quality of the data and ensuring the high representativeness of the sample [54].

### 3.2. Measurement of Key Variables

Explained variables: The food consumption structure is used as the explained variable in this study. Following Qiu et al. and Cui et al., the diet quality divergence (DQD) index serves as a more accessible proxy variable, facilitating its application and understanding for both policymakers and consumers [1,55]. The DQD index is an absolute divergence value (percent) representing the gap between real food consumption and recommended intake levels specified in the Chinese Food Guide Pagoda (CFGP). The CFGP provides the daily recommended intake of food categories for Chinese residents. The food categories selected in this study include seven types: staple foods, legumes, poultry and livestock products, aquatic products, eggs, milk and dairy products, and vegetables. It is important to note that the CFGP has updated the recommended intake for milk and dairy products, adjusting the 2016 guideline of over 300 g to a range of 300 to 500 g in the 2022 version. Additionally, the CFGP2022 does not provide specific recommendations for egg consumption. Therefore, this study utilizes the standards established in both the 2016 and 2022 versions. The data utilized in this analysis are derived from the “household staple food consumption” section of the CRRS questionnaire. Given that the food categories included in the questionnaire are limited to seven categories, slight adjustments were made based on the existing literature and the DQD formula. The formula for calculating the DQD index is presented as follows:

(1) $$X_{ik}=\frac{1}{30n}\times x_{ik}$$

(2) $${DQD}_{ik}=\frac{\left(\left|X_{ik}-R_{k}\right|\right)}{R_{k}}\times 100\%$$

(3) $${DQD}_{i}=\sum_{k=1}^{7}{DQD}_{ik}$$

where $X_{ik}$ is the daily per capita intake of food category k for household i. $x_{ik}$ is the total intake of food category k for household i over the past month. n is the number of household members eating at home. $R_{k}$ is the limit of the recommended intake for food category k. ${DQD}_{ik}$ is the diet quality divergence index of food category k for household i, computed as the absolute deviation between the average daily consumption $X_{ik}$ and corresponding recommendation $R_{k}$ in the Chinese dietary guidelines. When $X_{ik}$ > max$R_{k}$, $R_{k}$ = max$R_{k}$; when $X_{ik}$ < max$R_{k}$, $R_{k}$ = min$R_{k}$; and when min$R_{k}$ ≤ $X_{ik}$ ≤ max$R_{k}$, ${DQD}_{ik}$ = 0. ${DQD}_{i}$ is the total diet quality divergence index for household i, summing up all divergences for the seven food categories. The range of DQD is [0, +∞), and a smaller DQD index indicates better diet quality and vice versa. When DQD approaches 0, it means the respondent’s diet is fully consistent with the intake standards of Chinese dietary guidelines.

Explanatory variables: To assess digital literacy, this paper draws upon methodologies employed in prior research [22,56], taking into account the information skills, media application skills, production skills, and life skills of rural residents (See Table 1). An evaluation index system for digital literacy was constructed accordingly. A total score was generated for each subscale by using an entropy weight method, which assigns weights based on the intrinsic information content of the data, thereby minimizing the bias introduced by subjective factors. Higher scores indicate higher levels of digital literacy.

Mechanism variables: Residents’ food health awareness comprises two aspects—dietary health awareness and food safety awareness [45,46]. Four items were chosen to measure dietary health awareness, and five questions were used to assess food safety awareness (See Table 2). The equal weight method was employed to aggregate the values across all dimensions, resulting in the derivation of indices for dietary health awareness and food safety awareness among rural residents. A composite index closer to 1 reflects a higher level of awareness, while an index approaching 0 indicates a lower level of awareness.

Control variables: Based on the existing literature, we tried to control as many factors as possible influencing rural residents’ food consumption structure [37]. The control variables include the following: age, marital status, education level, employment status, income level, the number of children and elderly individuals in the household, and subjective perceptions of health.

### 3.3. Descriptive Statistics

Table 3 shows the definitions, mean, and standard deviation of the core variables in this paper. Overall, there is still a significant gap between the food intake of the participants and the recommended intake levels. Furthermore, as indicated by the mean value, the digital literacy level among rural residents is notably low. Regarding control variables, the majority of rural residents are aged 50 and above, with 91.2% being married and having an average education level of junior high school. A significant proportion, 74.3%, are employed full-time or part-time in agriculture, and 62.5% have been diagnosed with chronic diseases, such as hypertension and hyperlipidemia. The mean proportions of elderly individuals and children in rural households are 0.324 and 0.103, respectively, indicating a substantial elderly population. When rural residents are grouped according to income quintiles, the mean value is 2.920, suggesting a high prevalence of lower-middle-income individuals.

### 3.4. Regression Model

The model used to investigate the impact of digital literacy on rural residents’ food consumption structure is constructed as follows [6,30]:

(4) $${DQD}_{i}=\alpha_{0}+\alpha_{1}{DL}_{i}+\alpha_{2}{ConVar}_{i}+\eta_{j}+\varepsilon_{i}$$

where i denotes household; ${DQD}_{i}$ represents deviations in household food consumption structure. ${DL}_{i}$ represents the level of digital literacy of rural residents, which is the core explanatory variable. ${ConVar}_{i}$ represents a series of control variables. Additionally, provincial fixed effects $\eta_{j}$ are incorporated into the model to address differences in dietary habits across various regions. $\alpha_{0}$ represents a constant term. $\alpha_{1}$ reflects the change in food structure resulting from variations in rural residents’ digital literacy, which is the estimation coefficient that this study focuses on. $\varepsilon_{i}$ represents the random error term.

## 4. Analysis of Empirical Results

### 4.1. Baseline Regression Results

Building on the theoretical analysis presented above, this paper empirically examines the relationship between rural residents’ digital literacy and their food consumption structure. Table 4 displays the results of the ordinary least squares (OLS) regression. Columns (1) and (2) present the overall regression outcomes for digital literacy both without and with control variables, respectively. The results indicate that, after accounting for provincial fixed effects, the impact of rural residents’ digital literacy on deviations in their food consumption structure is statistically significant at the 1% level, with a negative coefficient. This suggests that as digital literacy improves, the deviations in residents’ food consumption structure diminish.

### 4.2. Robustness Tests

#### 4.2.1. Replace Explanatory Variables

To ensure the robustness of the aforementioned results, this paper substitutes digital literacy with internet usage [37]. This variable is derived from the item “household Internet access device” in the CRRS questionnaire. Specifically, if family members utilize mobile devices or computers to access the internet, the variable is assigned a value of 1; otherwise, it is 0. From an individual perspective, the development of the internet can benefit rural residents through its inclusive and shared nature, enhancing their awareness of healthy dietary practices and improving their food consumption structure. The regression results for the substituted variable presented in column (1) of Table 5 indicate that internet usage significantly decreases the deviation in residents’ food consumption structure. Notably, these results remain significant even after replacing the explanatory variables, thereby confirming the robustness of the initial findings.

#### 4.2.2. Replace Explained Variables

Dietary diversity is a fundamental principle of balanced dietary patterns, and the Dietary Diversity Score (DDS) has been utilized as a proxy for DQD in the regression analysis. Based on the food classification outlined in the CFGP2022, each food category consumed earns one point, with scores ranging from 0 to 7. A higher score indicates greater dietary diversity and balance. The results presented in Table 5, column (2), reveal that digital literacy significantly enhances dietary diversity among rural residents. Furthermore, these findings remain statistically significant even after substituting the explanatory variables, thereby demonstrating the robustness of the initial results.

#### 4.2.3. Shrink the Sample

According to the 45th “Statistical Report on China’s Internet Development” released by CNNIC for March 2020, the proportions of internet users aged 20–29, 30–39, and 40–49 were 21.5%, 20.8%, and 17.6%, respectively, while those aged 50 and above constituted 16.9% of the total internet user population (CNNIC, 2024 [10]). After data cleaning, the sample for this study spans ages 23 to 89 years. Given improvements in living conditions, advancements in medical standards, and enhanced health awareness, life expectancy has been increasing [59]. The World Health Organization classifies individuals aged 60 to 74 as “young older adults” and those aged 75 to 89 as “older adults.” Additionally, the United Nations typically defines older persons as individuals aged 60 or 65 and above; in some developed Western countries, this threshold is set at over 65 years. Therefore, this paper conducts separate regressions for samples under 75 years of age and under 65 years. The results indicate that the regression outcomes remain significant even after reducing the sample size, thereby confirming the robustness of the initial findings. Detailed results are presented in columns (3) and (4) of Table 5.

#### 4.2.4. Endogeneity Analysis: Instrumental Variable Estimation

OLS regression may encounter endogeneity issues stemming from omitted variable bias and bidirectional causality. This paper employs the instrumental variable method to further assess robustness, selecting “the average level of digital literacy at the village level excluding respondents” and “internet familiarity” as instrumental variables for digital literacy [16]. The rationale for selecting the first instrumental variable is that individual digital literacy may be influenced by other village members; however, there exists no direct correlation between the digital literacy level of the village and the food consumption structure of rural households. The justification for the second instrumental variable is twofold: firstly, effective digital infrastructure is a prerequisite for fostering and enhancing digital literacy, as rural residents require basic equipment to fully utilize the benefits of their digital skills. Secondly, the degree of online familiarity does not have a direct relationship with the food consumption structure. Consequently, these instrumental variables meet both the correlation and exogeneity conditions.

Table 6 presents the regression results obtained using the two-stage least squares (2SLS) method. The results of the identification and weak instrument tests in the CRRS indicate a *p*-value of 0.000 for the Kleibergen–Paap rk LM statistic in the first stage of the instrumental variable analysis, with the Cragg–Donald Wald F-statistic at 42.63 and the Kleibergen–Paap rk Wald F-statistic at 44.30. All of these values exceed the threshold of 10, confirming that the instrumental variables do not suffer from issues of identification or weakness, and that the correlation research hypothesis is upheld. The instrumental variables selected in this study are valid. Additionally, the results from the second stage align with the OLS regression findings. This consistency reinforces the conclusion that the digital literacy of rural residents significantly enhances their food consumption structure, demonstrating strong robustness in the results.

### 4.3. Heterogeneity Analysis

#### 4.3.1. Heterogeneity Analysis Based on Region

Considering the differences in dietary culture and habits between northern and southern China, the role of digital literacy may vary, leading to significant disparities in the utility that residents derive from their digital skills. Consequently, this paper primarily analyzes regional heterogeneity in the relationship between digital literacy and food consumption structure, exploring the differences in the efficacy of digital skills across regions with distinct dietary cultures and habits. The analysis categorizes regions into northern and southern areas to investigate the impact of digital literacy on residents’ food consumption structure. The results presented in column (1) of Table 7 indicate that the digital benefits gained through digital literacy are significantly greater for residents in the southern region than for those in the northern region. This may be due to the fact that the southern regions are more economically developed, with a higher degree of marketization and a well-established food supply chain. As a result, improvements in digital literacy are more likely to lead to a greater demand for high-quality and diverse foods. In contrast, some northern regions experience slower economic development, lower levels of marketization, and less developed food supply chains. In these areas, the increase in digital literacy has yet to result in an improvement in the quality of food consumption.

#### 4.3.2. Heterogeneity Analysis Based on Education Level

This study categorizes the education level of household heads into two groups: low education level and high education level. “Low education” refers to rural households whose highest level of education is junior high school or below, while “high education” pertains to those who have completed high school or obtained a higher degree. The results presented in column (2) of Table 7 indicate that digital literacy has a significant negative impact on DQD among rural residents with low education levels, while its effect on DQD among rural residents with high education levels does not pass the significance test. This may be attributed to the limited information access channels and narrower knowledge base regarding asset allocation among low-educated rural residents. Digital literacy appears to notably enhance their ability to acquire information and expand their understanding of healthy dietary practices, thus facilitating the optimization of their food consumption structures. In contrast, rural households with high education levels possess a higher overall knowledge base, resulting in a non-significant effect of digital literacy on deviations in their food consumption structure. Consequently, digital literacy serves to mitigate disparities between different educational groups, thereby improving the structural biases in food consumption among individuals with lower education levels.

#### 4.3.3. Heterogeneity Analysis Based on Age

Based on the age of household heads, the sample of rural residents was categorized into two groups: the youth group (ages 18–40) and the middle-aged and elderly group (ages 41 and above). The results presented in column (3) of Table 7 indicate that digital skills do not have a significant impact on DQD among young rural residents; however, they exert a significant negative impact on deviations in food consumption structures among middle-aged and elderly rural residents. This may be attributed to the fact that younger rural residents possess more information channels and knowledge reserves, rendering the influence of digital literacy on their food consumption structures insignificant. In contrast, middle-aged and elderly rural residents often face challenges in information acquisition, and digital literacy can significantly broaden their access to information channels, thereby optimizing their food consumption structures.

## 5. Mechanism Analysis

According to the preceding analysis, digital literacy primarily improves the food consumption structure of rural residents by enhancing their awareness of dietary health and food safety. Consequently, this paper will empirically investigate the specific mechanisms underlying this influence. The relevant indicators are outlined in Table 2. Following the methodology for testing influence mechanisms proposed by Jiang [60], the following model is constructed:

(5) $$M_{i}=\alpha_{3}+\alpha_{4}{DL}_{i}+\alpha_{5}{ConVar}_{i}+\eta_{j}+\varepsilon_{i}$$

where $M_{i}$ denotes the mechanism variables, i.e., dietary health awareness and food safety awareness; ${DL}_{i}$ is the level of digital literacy of rural residents; ${ConVar}_{i}$ denotes the series of control variables mentioned above; $\alpha_{3},\;\alpha_{4},\;\alpha_{5}$ are the parameters to be estimated. If $\alpha_{4}$ is significant, it indicates that the influencing mechanism is indeed present.

### 5.1. Mechanism Analysis of Dietary Health Awareness

The results are presented in Table 8. Columns (1) to (5) illustrate the impact of digital literacy on dietary health awareness. The findings indicate that higher levels of digital literacy correlate with increased awareness of healthy dietary practices among rural residents. Moreover, when examining the individual categorical indicators of dietary health awareness, it was revealed that digital literacy positively influences rural residents’ recognition of health preservation, sugar control, salt control, and oil control, with all effects achieving statistical significance at the 1% level. This suggests that enhancing the digital literacy of rural residents can improve their awareness of dietary health, thereby optimizing their food consumption structures.

### 5.2. Mechanism Analysis of Food Safety Awareness

The results of food safety awareness are presented in Table 9. Columns (1) to (6) illustrate the impact of digital literacy on food safety awareness. The findings indicate that higher levels of digital literacy are associated with increased food safety awareness among rural residents. Furthermore, when examining the individual categorical indicators of food safety awareness, it was found that digital literacy positively influences awareness regarding chemical residues, additives, counterfeit foods and biotech foods, all of which achieved statistical significance at the 1% or 5% level. This suggests that enhancing the digital literacy of rural residents can significantly improve their awareness of food safety and optimize their food consumption structures.

## 6. Discussion

Optimizing the food consumption structure is of paramount importance in safeguarding residents’ nutritional health, promoting economic and social development, and addressing food safety challenges. This study utilizes the 2020 CRRS data to investigate the impact and underlying mechanisms of digital literacy on the food consumption structure of rural residents, with the goal of providing valuable insights for advancing the optimization of food consumption patterns among rural residents.

In the field of digital economy research, the existing literature predominantly addresses two dimensions: At the macro level, research primarily explores the impact of the digital economy on consumption from the perspectives of e-commerce development, digital inclusive finance, and the upgrading of traditional industries [32,61,62]. At the micro level, studies typically utilize micro-survey data to examine the influence of internet usage, e-commerce, mobile payments, and digital literacy on residents’ consumption levels, consumption patterns, and consumption inequality, with findings indicating that digital technology and digital literacy play a significant role in promoting consumption upgrading [31,32]. However, existing research has yet to sufficiently address the relationship between digital literacy and food consumption structure. This study empirically examines the relationship between digital literacy and the food consumption structure of rural residents, thereby contributing to the expansion of research on the factors influencing food consumption.

In terms of data, previous studies have often relied on historical datasets, such as the China Health and Nutrition Survey (CHNS), which has limitations in terms of timeliness [63]. In contrast, this study utilizes the most recent data from the 2020 CRRS, whose sample characteristics more accurately reflect the current food consumption patterns of rural residents in China, thereby significantly enhancing the timeliness and policy relevance of the research findings. Additionally, past research has two limitations: on one hand, macro-level data analysis struggles to capture individual consumption characteristics [64]; on the other hand, micro-level studies have often overly relied on a single indicator—food consumption expenditure—focusing on the quantity of food consumption while overlooking the quality dimension [65]. This study addresses these gaps by introducing dietary quality measures such as the DQD index and the DDS, thereby validating the positive impact of digital literacy on optimizing food consumption structure from both quantity and quality perspectives. Our findings suggest that digital literacy can improve the dietary structure of rural residents, providing empirical evidence for the role of digital literacy in optimizing food consumption patterns.

This study examines the heterogeneity of digital literacy. The results from the heterogeneity analysis suggest that digital literacy has a more pronounced effect on the deviation in food consumption structure among rural residents in southern regions. This finding aligns with stratification theory, which posits that regions with more advanced economic development and technological infrastructure are better positioned to benefit from the digital economy [57]. Furthermore, enhancing digital literacy has a significantly positive impact on the food consumption quality of individuals with lower levels of education and older adults. This indicates that improving digital literacy can reduce health disparities associated with education and age, thus contributing new empirical evidence to social stratification theory.

Moreover, from the perspective of behavioral mechanisms, this study elucidates the underlying pathway through which digital literacy influences food consumption structure, drawing on the HBM. Specifically, through digital media, rural residents gain a more intuitive understanding of food safety information and nutritional health knowledge. This heightened awareness leads to an increase in health consciousness, ultimately motivating individuals to adopt a more scientifically informed dietary structure. The finding offers a novel theoretical perspective for understanding how digital literacy reshapes the food consumption concepts and structures of rural residents.

This study also has several limitations that require further refinement in future research. First, the measurement system for digital literacy requires further optimization. With the rapid development of the digital economy, the concept of digital literacy is continuously evolving, encompassing various dimensions such as digital skills, capabilities, and attitudes. However, due to constraints in research duration and budget, this study has limitations in constructing the dimensions of digital literacy measurement. Future research should consider incorporating more comprehensive and multidimensional measurement frameworks. Second, this study also has limitations regarding its temporal dimension. By relying on cross-sectional data, the research only analyzes the short-term effects of digital literacy, making it difficult to assess its long-term dynamic impacts. Future studies could use longitudinal data to provide a more comprehensive view of the ongoing impact of digital literacy on changes in dietary behaviors among rural residents.

## 7. Conclusions and Policy Implications

### 7.1. Conclusions

With the rapid advancement of digital technology, enhancing the digital literacy of rural residents and optimizing their food consumption patterns has emerged as a crucial strategic initiative to promote healthy dietary practices and advance national nutrition standards. Drawing on the CRRS, this paper investigates the influence of digital literacy on the food consumption structures of rural residents and further explores the underlying mechanisms in terms of awareness. The findings indicate that, firstly, the digital literacy of rural residents significantly enhances their food consumption structures. This result remains robust even after addressing endogeneity concerns through various tests and instrumental variable estimation. Secondly, the heterogeneity analysis based on regional differences, educational attainment, and age reveals that improvements in digital literacy are particularly beneficial for enhancing the food consumption structures of residents in southern regions. Additionally, digital literacy has a pronounced impact on mitigating the disparities in food consumption structures among individuals with lower educational levels, as well as among middle-aged and elderly populations, effectively reducing inter-group differences. Finally, this study examines the internal mechanisms linking digital literacy to the optimization of food consumption structures through the lenses of dietary health awareness and food safety awareness. The results underscore that both dietary health awareness and food safety awareness serve as significant mechanisms through which digital literacy positively influences the food consumption patterns of rural residents.

### 7.2. Policy Implications

Based on the primary research conclusions presented in this paper, the following recommendations are proposed:

First, the foremost priority is to expedite the construction of “Digital China”, which fundamentally involves the comprehensive optimization and enhancement of digital access quality in rural and underdeveloped areas. Although the penetration rate of internet infrastructure has been steadily increasing in recent years, significant disparities in digital literacy levels and the capacity to utilize digital technologies persist across various regions and social groups. Thus, it is imperative to adopt more vigorous measures to accelerate the development of digital infrastructure, with a particular focus on bolstering support for and promoting the application of digital technologies in underdeveloped regions. Achieving simultaneous progress in both infrastructure enhancement and quality management will effectively mitigate the digital divide among different regions and demographic groups, ensuring that the benefits of digital advancement are distributed more widely and equitably among all citizens.

Second, we must enhance the dissemination of digital and nutritional health knowledge while conducting relevant training and educational programs to improve digital skills among rural residents. It is vital to tailor the delivery of digital knowledge and nutritional education to the food consumption levels, preferences, and knowledge backgrounds of low-educated groups, middle-aged individuals, and elderly populations in rural areas. For groups reluctant to adopt digital technology, the government should introduce incentive mechanisms, such as social recognition or small rewards, to encourage continued participation. By implementing precise and differentiated approaches to the dissemination of digital knowledge and nutritional health information, we can promote the upgrading of food consumption among rural residents towards higher quality and greater diversification.

Third, from the perspective of food supply, digital technologies should be leveraged to expand food supply channels and diversify the variety of food available in rural areas. Improvements in rural residents’ digital literacy are necessary to empower them to utilize these technologies effectively, thereby enhancing their food consumption structures.

Fourth, it is crucial to harness the positive impact of digital technology on the accumulation and dissemination of health information among rural residents. By fostering awareness of dietary health and food safety, we can achieve sustained and effective optimization of the food consumption structures of rural populations.

Fifth, to systematically enhance the digital literacy of rural residents and optimize their food consumption structure, this study recommends establishing a comprehensive intervention mechanism led by the government, with collaboration across multiple departments and active social participation. Specifically, a collaborative governance platform should be created, spearheaded by the health and wellness department, and involving key sectors such as agriculture, rural affairs, education, civil affairs, and industry and information technology. This platform would integrate policy resources, optimize service delivery, and innovate intervention models, fostering an organic combination of digital literacy training and dietary nutrition health education.

## Figures and Tables

**Table 1:** Digital literacy indicator system.

Standard Floor	Index Description
Information skills [8,57]	Browsing news online through mobile phones or the internet: Yes = 1; No = 0
	Following news events through mobile phones or the internet: Yes = 1; No = 0
	Obtaining employment and entrepreneurship information through mobile phones or the internet: Yes = 1; No = 0
	Purchasing data analysis services through mobile phones or the internet: Yes = 1; No = 0
Media application skills [57]	Engaging in recreational activities through mobile phones or the internet: Yes = 1; No = 0
	Accessing information about recreational activities through mobile phones or the internet: Yes = 1; No = 0
Production skills [57,58]	Obtaining production guidance information through mobile phones or the internet: Yes = 1; No = 0
	Using mobile phones or the internet for product transactions: Yes = 1; No = 0
Life skills [58]	Acquiring life-related knowledge through mobile phones or the internet: Yes = 1; No = 0
	Engaging in communication and social activities via online platforms (e.g., WeChat, Weibo, QQ, Zhihu, Douban): Yes = 1; No = 0
	Utilizing mobile phones or the internet for learning and educational purposes: Yes = 1; No = 0

**Table 2:** Selection of food health awareness indicators.

Primary Index	Secondary Index	Index Description
Dietary health awareness	Health awareness	Whether there is a conscious effort to learn about health or wellness knowledge: Yes = 1; No = 0
	Awareness of sugar control	Whether there is a conscious effort to manage sugar intake: Yes = 1; No = 0
	Awareness of salt control	Whether there is a conscious effort to manage salt intake: Yes = 1; No = 0
	Awareness of oil control	Whether there is a conscious effort to manage oil intake: Yes = 1; No = 0
Food safety awareness	Chemical residues	Concerned about issues related to chemical residues such as pesticides and insecticides: Yes = 1; No = 0
	Additive	Concerned about the use of additives such as preservatives and colorants: Yes = 1; No = 0
	Food spoilage	Concerned about food spoilage issues: Yes = 1; No = 0
	Counterfeit foods	Concerned about issues related to adulteration and the presence of substandard or counterfeit food products: Yes = 1; No = 0
	Biotech foods	Concerned about the use of biotechnology, including genetically modified organisms: Yes = 1; No = 0

**Table 3:** Descriptive Statistics.

Variables	Definitions	Mean	SD
Explained variables			
Diet quality divergence (DQD) index	Diet quality divergence index: sum divergences over seven food categories to measure overall diet quality (100%)	564.526	523.877
Explanatory variables			
Digital literacy	Digital literacy measurement index score: 0~1	0.183	0.147
Mechanism variables			
Dietary health awareness	Dietary health awareness measurement index score: 0~1	0.304	0.230
Food safety awareness	Food safety awareness measurement index score: 0~1	0.571	0.406
Control variables			
Age	20–29 years = 1; 30–39 years = 2; 40–49 years = 3; 50–59 years = 4; 60–69 years = 5; 70–79 years = 6; 80–89 years = 7	4.028	1.197
Marital status	Not married = 0; Married = 1	0.912	0.284
Educational attainment	Number of years of education completed	7.937	3.293
Employment status	Whether engaged in full-time agricultural work: No = 0; Yes = 1	0.743	0.437
Subjective health	Whether diagnosed with a chronic disease: No = 0; Yes = 1	0.625	0.484
Income level	Low income = 1; Lower-middle income = 2; Middle income = 3; Upper-middle income = 4; High income = 5	2.920	1.397
Elderly proportion	Number of family members aged 60 and above who have dined at home in the past month	0.324	0.385
Children proportion	Number of children aged 12 and under who have dined at home in the past month	0.103	0.170

**Table 4:** OLS regression of digital literacy and food consumption structure.

Variables	(1)	(2)
Digital literacy	−205.153 ***	−170.160 **
	(51.964)	(69.084)
Age (the group aged 29 and below is used as the reference group)		
Aged 30–39		23.381
		(53.692)
Aged 40–49		25.304
		(51.422)
Aged 50–59		69.494
		(53.316)
Aged 60–69		32.906
		(52.713)
Aged 70–79		22.861
		(56.715)
Aged 80 and above		10.157
		(108.612)
Marital status		−104.420 ***
		(33.226)
Educational attainment		−1.542
		(3.640)
Employment status		39.940 **
		(17.074)
Subjective health		−0.436
		(20.968)
Children proportion		−254.584 ***
		(63.165)
Elderly proportion		13.824
		(31.280)
Income level (the low-income group is used as the reference group)		
Lower-middle income		26.362
		(23.979)
Middle income		6.190
		(23.715)
Upper-middle income		5.340
		(25.026)
High income		63.812
		(39.315)
Provincial fixed effect	YES	YES
Constant	1132.222 ***	1155.232 ***
	(51.379)	(77.836)
Number	2827	2827
R-squared	0.147	0.161

Note: ** and *** denote significance at the 0.05 and 0.01 level, respectively. Robust standard errors are in parentheses.

**Table 5:** Robustness analysis.

Variables	(1)	(2)	(3)	(4)
	Replace Explanatory Variables	Replace Explained Variables	Shrink the Sample (<75)	Shrink the Sample (<65)
Internet usage	−70.685 **			
	(31.381)			
Digital literacy		0.339 **	−168.048 **	−169.696 **
		(0.160)	(82.470)	(71.045)
Control variable	YES	YES	YES	YES
Provincial fixed effect	YES	YES	YES	YES
Constant	1182.962 ***	5.205 ***	1164.433 ***	1169.271 ***
	(80.161)	(0.179)	(89.521)	(82.929)
Number	2827	2827	2172	2711
R-squared	0.160	0.166	0.152	0.157

Note: ** and *** denote significance at the 0.05 and 0.01 level, respectively. Robust standard errors are in parentheses.

**Table 6:** Endogeneity analysis results.

Variables	(1)
Second stage	
Digital literacy	−1217.515 ***
	(440.179)
Control variable	YES
Provincial fixed effect	Yes
Constant	1340.751 ***
	(114.751)
Number	2827
R-squared	0.096
First stage	
Instrumental variable I	0.158 ***
	(0.038)
Instrumental variable II	0.026 ***
	(0.003)
Cragg–Donald Wald F-statistic	42.63

Note: *** denotes significance at the 0.01 level. Robust standard errors are in parentheses.

**Table 7:** Heterogeneity analysis results.

Variables	(1) Region		(2) Education		(3) Age	
	Northern Region	Southern Region	Low Education	High Education	Youth	Middle-Aged and Elderly
Digital literacy	−68.238	−153.255 **	−427.506 ***	−43.576	−40.955	−212.705 **
	(120.475)	(61.169)	(101.762)	(84.752)	(86.767)	(90.135)
Control variable	YES	YES	YES	YES	YES	YES
Provincial fixed effect	YES	YES	YES	YES	YES	YES
Constant	928.327 ***	494.262 ***	1265.603 ***	948.856 ***	1108.325 ***	1213.739 ***
	(150.370)	(47.644)	(182.746)	(203.607)	(150.890)	(79.981)
Number	1508	1319	1075	1752	511	2316
R-squared	0.022	0.026	0.230	0.133	0.224	0.157

Note: ** and *** denote significance at the 0.05 and 0.01 level, respectively. Robust standard errors are in parentheses.

**Table 8:** Mechanism analysis results for dietary health awareness.

Variables	(1)	(2)	(3)	(4)	(5)
	Dietary Health Awareness	Health Awareness	Sugar Control	Salt Control	Oil Control
Digital literacy	0.297 ***	0.467 ***	0.253 ***	0.276 ***	0.190 ***
	(0.057)	(0.069)	(0.070)	(0.068)	(0.070)
Control variable	YES	YES	YES	YES	YES
Provincial fixed effect	YES	YES	YES	YES	YES
Constant	0.422 ***	0.053	0.581 ***	0.572 ***	0.482 ***
	(0.066)	(0.083)	(0.077)	(0.073)	(0.077)
Number	2827	2827	2827	2827	2827
R-squared	0.097	0.097	0.068	0.073	0.076

Note: *** denotes significance at the 0.01 level. Robust standard errors are in parentheses.

**Table 9:** Mechanism analysis results for food safety awareness.

Variables	(1)	(2)	(3)	(4)	(5)	(6)
	Food Safety Awareness	Chemical Residues	Additives	Food Spoilage	Counterfeit Foods	Biotech Foods
Digital literacy	0.133 ***	0.178 **	0.195 ***	0.039	0.148 **	0.104 **
	(0.036)	(0.072)	(0.068)	(0.073)	(0.066)	(0.042)
Control variable	YES	YES	YES	YES	YES	YES
Provincial fixed effect	YES	YES	YES	YES	YES	YES
Constant	0.315 ***	0.343 ***	0.374 ***	0.442 ***	0.279 ***	0.136 ***
	(0.046)	(0.086)	(0.083)	(0.087)	(0.080)	(0.047)
Number	2827	2827	2827	2827	2827	2827
R-squared	0.082	0.056	0.070	0.033	0.040	0.054

Note: ** and *** denote significance at the 0.05 and 0.01 level, respectively. Robust standard errors are in parentheses.

## Data Availability

Restrictions apply to the availability of these data. The data were obtained from the Comprehensive Survey on Rural Revitalization and China Rural Revitalization Survey (CRRS) database (Project Numbers: GQDC202017; GQDC2022020; 2024ZDDC001), the Major Economic and Social Investigation Project funded by the Chinese Academy of Social Sciences. These data are available for research purposes only. Users need to apply in the name of an institution. The datasets are available upon reasonable request and with permission of the Rural Development Institute, Chinese Academy of Social Sciences.
